# Annular elastolytic giant cell granuloma resolution after pulsed dye laser

**DOI:** 10.1016/j.jdcr.2025.02.007

**Published:** 2025-03-07

**Authors:** Devyn Zaminski, Michelle Juarez, Stephanie Eichman, Daniel R. Mazori, Michael Lee, Lisa Akintilo, Avrom S. Caplan

**Affiliations:** a The Ronald O. Perelman Department of Dermatology, New York University Grossman School of Medicine, New York, New York; b Division of Rheumatology, Department of Medicine, New York University Grossman School of Medicine, New York, New York; c Dermatology Service, Bellevue Hospital, New York, New York

**Keywords:** actinic granuloma, annular elastolytic giant cell granuloma, pulsed dye laser, sun exposure

## Introduction

Annular elastolytic giant cell granuloma (AEGCG), or actinic granuloma, is a rare, idiopathic skin condition characterized by pruritic or asymptomatic papules that become annular plaques with erythematous, raised borders, and a hypopigmented center.^1^^,^^2^ Lesions may predominate on sun-exposed areas^1^^,^^2^ and affect self-consciousness. Presentation typically occurs between ages 30 and 75 years, with similar rates across genders, although some reports indicate a female predominance and possible childhood onset.^2^ Associated systemic diseases include diabetes mellitus, solid organ malignancies, sarcoidosis, and rarely hematologic disorders.^2^ AEGCG pathogenesis is poorly understood, but may involve chronic inflammation from photo-induced elastin damage, exposing antigens that trigger an immune response.^1^ Alternatively, idiopathic granulomatous inflammation may cause elastin injury, rather than actinic radiation.^3^ Some consider AEGCG a photo-distributed form of granuloma annulare. Histology demonstrating 3 distinct regions is diagnostic: a peripheral zone with prominent elastosis, papillary/mid-dermal region with histiocytic and giant cell inflammatory infiltrate with possible elastophagocytosis, and central area with few or absent elastic fibers.^1^ AEGCG can be self-limited but is often chronic, with limited literature guiding management.^2^ First line therapies include cryotherapy, topical/intralesional steroids, and topical immunomodulators.^2^^,^^4^ Refractory cases can be managed with antimalarials, retinoids, systemic steroids, methotrexate, dapsone, cyclosporine, or tumor necrosis factor-inhibitors, although data are limited.^2^^,^^4^^,^^5^ Pulse dye laser (PDL) is widely used to treat a variety of skin conditions, predominantly cutaneous vascular disorders, with common side effects of procedural discomfort and transient erythema or bruising.^6^ PDL is rarely reported in AEGCG management, with only 2 prior case reports combining PDL with another laser or injection.^6^, ^7^, ^8^ Here, we present a 53-year-old woman with inactive pulmonary sarcoidosis diagnosed with AEGCG via punch biopsy. After failing multiple therapies, her lesions exhibited rapid and significant improvement after one PDL treatment alone.

## Case report

**Fig 1:**
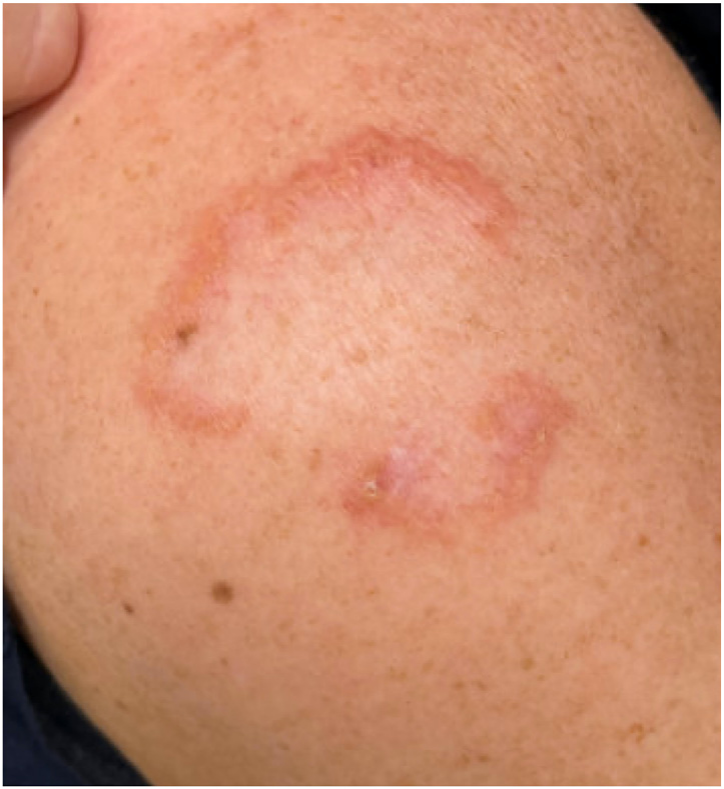
Annular elastolytic giant cell granuloma lesion as an annular plaque with raised erythematous boarders, located on the patient’s left deltoid, appearing before pulse dye laser therapy.

A 53-year-old woman presented to Dermatology for annular plaques on her forehead, cheeks, shoulders, and back for 2 years (Fig 1). Her medical history included pulmonary sarcoidosis, hyperlipidemia, thalassemia minor, and hypothyroidism. She regularly followed with rheumatology and cardiology who confirmed inactive systemic sarcoidosis. Chest x-rays since 2015 were reportedly normal, and neither electrocardiogram nor echocardiogram raised concern for cardiac sarcoidosis. She never required therapy for pulmonary sarcoidosis but was referred to Dermatology. The differential diagnosis at the time of evaluation included cutaneous sarcoidosis, AEGCG, and granuloma annulare. Previous skin treatments included hydroxychloroquine, methotrexate, leflunomide, adalimumab, and tofacitinib. Her records documented intolerance to methotrexate (25 mg weekly, 6 months) due to nausea and leflunomide (20 mg daily, 4 months) due to alopecia and diarrhea. Adalimumab led to partial improvement (40 mg every 2 weeks, 17 months), but was discontinued in favor of tofacitinib (11 mg extended release daily, 2 months). Tofacitinib resulted in improvement although was discontinued because of muscle cramping, so adalimumab was restarted. On referral, the patient remained on hydroxychloroquine (400 mg daily, years) and adalimumab (40 mg biweekly, 3 months).

**Fig 2:**
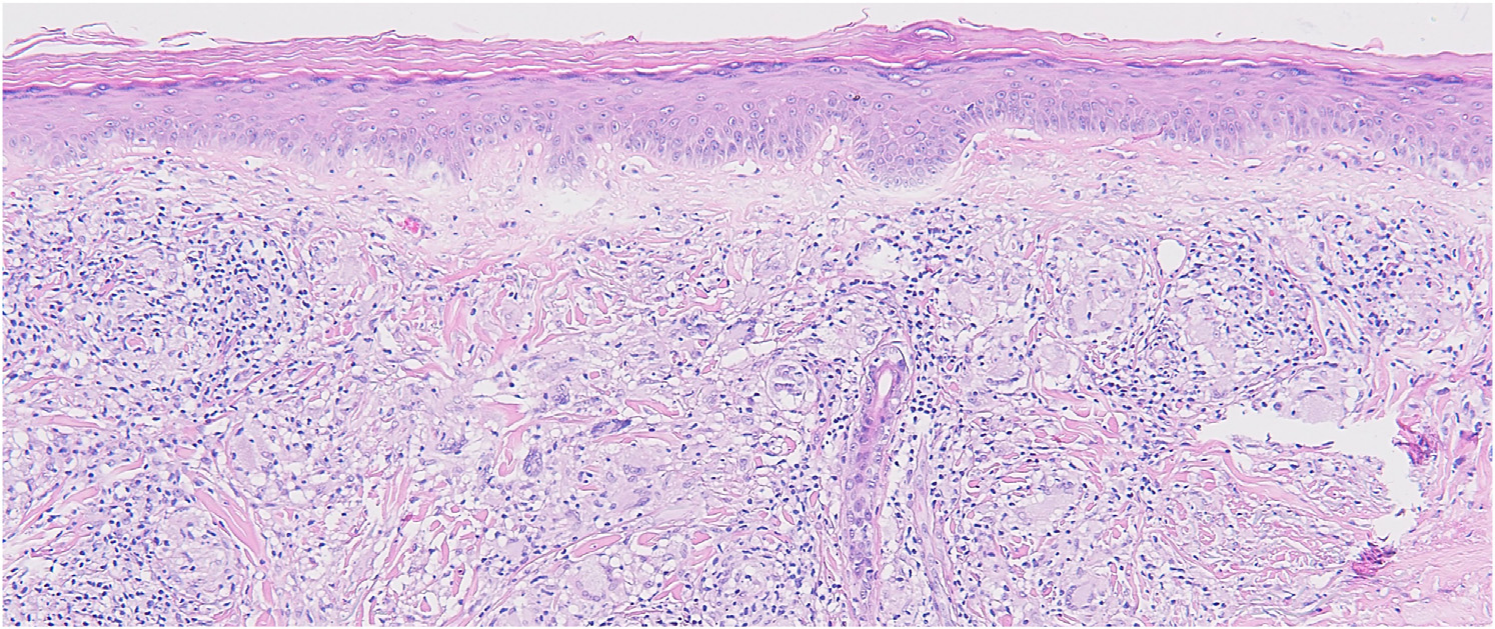
Skin biopsy of annular elastolytic giant cell granuloma demonstrating interstitial and nodular infiltrates of mono-/multinucleated histiocytes containing degenerated elastic fibers, areas of histiocytes palisading around mucin, and admixed perivascular lymphocytic infiltrate by hematoxylin-eosin stains (original magnification: ×10). There was no evidence of microorganisms.

**Fig 3:**
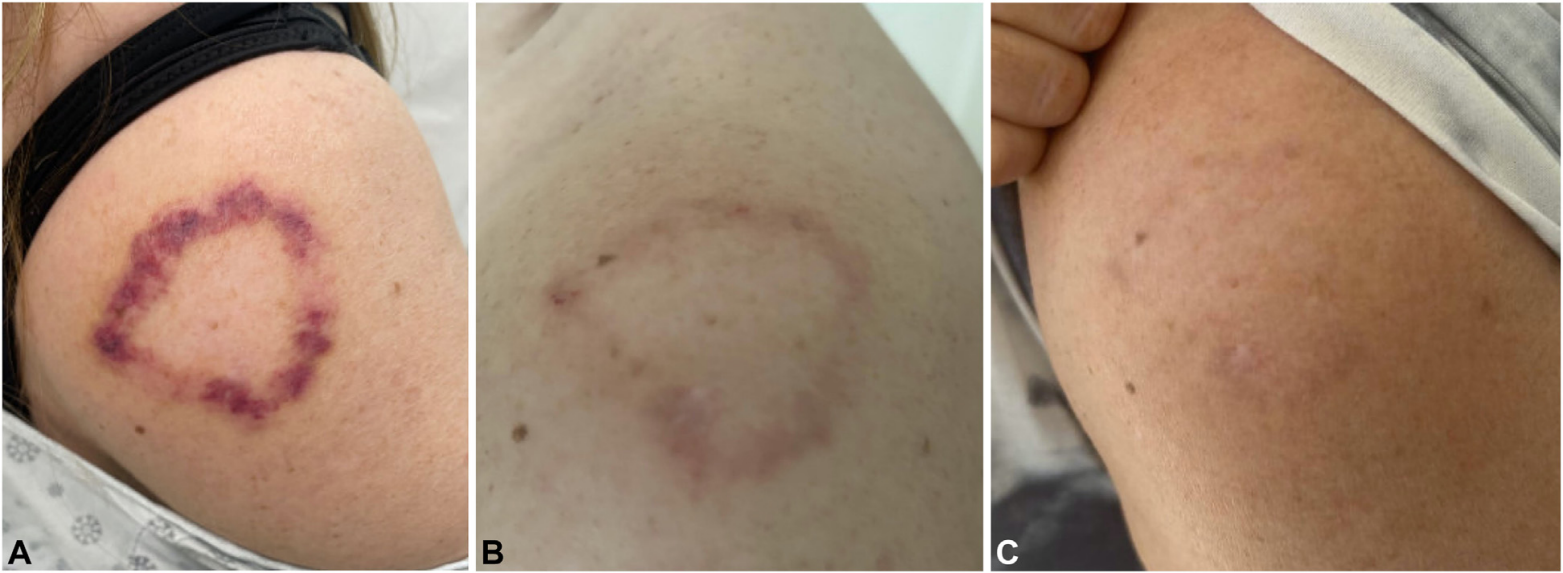
Annular elastolytic giant cell granuloma progressive resolution (**A**) 2 days, (**B**) 10 days, and (**C**) 1 month after pulse dye laser treatment.

A 4-mm punch biopsy of the left shoulder lesion revealed an interstitial and nodular infiltrate of mono-/multinucleated histiocytes containing degenerated elastic fibers, histiocytes palisading around mucin, admixed perivascular lymphocytic infiltrate, with no evidence microorganisms (Fig 2). Given her age, gender, and lesion morphology, a diagnosis of AEGCG was made. Hemoglobin A1c was 5.6%, indicating no diabetes. Over the following 2.5 years, the patient’s disease remained refractory to therapies including topical tacrolimus (0.1%, 16 months), intralesional kenalog, and topical tofacitinib (22 months). Hydroxychloroquine was continued, and adalimumab (additional 18 months), increased to weekly dosing, did not resolve her disease. Dapsone (max dose 150 mg, 4 months, caused anemia), pentoxifylline (400 mg 3 times a day, 3 days, caused gastrointestinal side effects), and upadacitinib (30 mg every other day, 1.5 months, caused muscle cramping and creatine kinase elevations) were trialed. She was subsequently referred for PDL and received 1 treatment on her left shoulder (595 nm wavelength, pulse width of 0.45 ms, fluence of 5 J/cm^2^, 10 mm spot size), resulting in rapid, drastic resolution of her chronic lesion (Fig 3). At that time, her regimen included hydroxychloroquine (400 mg), topical ruxolitinib (1.5%, 10 weeks), and periodic intralesional kenalog injections (8 total, most recently 3 months prior: 0.9 cc of 5 mg/mL in the forehead, nose, and shoulder). PDL therapy was well-tolerated, with self-limited erythema and crusting in the weeks following treatment. Five months post-PDL, our patient experienced sustained resolution of her AEGCG lesion without recurrence or further treatment.

## Discussion

We present a 53-year-old woman with AEGCG who failed multiple therapies before finally achieving resolution with PDL. AEGCG is rare, and evidence to define therapeutic approaches is lacking. PDL deploys selective photothermolysis, targeting hemoglobin to induce selective destruction of dermal vasculature. Therefore, PDL is hypothetically beneficial in AEGCG by reducing microcirculation in dermal vessels; however, PDL could also augment AEGCG by contributing photo-damage, a possible antigenic trigger of granulomatous inflammation.^1^^,^^6^ One case report proposed this theory when describing how PDL therapy, treating facial vascular abnormalities on a middle-aged woman, resulted in AEGCG formation from possible laser-induced actinic and/or heat damage.^9^

In addition to photothermolysis, PDL has been shown to induce anti-inflammatory cytokine production and collagen remodeling.^6^^,^^10^ In AEGCG, PDL could have reduced inflammation from damaged elastin, which are thought to underlie its pathogenesis.^1^^,^^4^ Further, in the context of collagen remodeling, PDL may have had a similar beneficial effect on elastin, contributing to its success in treating AEGCG.^10^ Regardless, 2 patients with refractory AEGCG were effectively treated using PDL, although combined with either a fractioned CO_2_ laser or intralesional polydeoxyribonucleotide (Table I).^7^^,^^8^

**Table I:** Summary of 3 successful therapy regimens for treating annular elastolytic giant cell granuloma that include PDL therapy; our patient and 2 other documented case reports

Full therapy regimen	Our patient: PDL, HCQ, topical ruxolitinib, intralesional steroid	Mamalis et al^7^: PDL, CO_2_ laser	Kim et al^8^: PDL, intralesional polydeoxyribonucleotide
No. PDL Txs	1	13	3
Latency between PDL tx (wks)	N/A	6	8
Wavelength (nm)	595	-	595
Pulse width (ms)	0.45	0.5-10.0	6
Spot size (mm)	10	7	-
Fluence (J/cm^2^)	5	12.5 then 7	8
Cryogen	30/20	-	-
Location	Left shoulder	Hands, arms	Face

*CO*_*2*_, Carbon dioxide; *HCQ*, hydroxychloroquine; *N/A*, not applicable; *PDL*, pulsed dye laser; *Tx*, treatment.

The dual nature of PDLs possible effect, both anti-inflammatory with extracellular matrix remodeling versus potentially photo-damaging, suggests a narrow therapeutic window for AEGCG treatment. Our patient achieved significant clinical improvement of her refractory AEGCG after one PDL treatment, underscoring its therapeutic potential. Unlike both aforementioned cases, our patient did not require combination therapy with another laser or injection.^7^^,^^8^ Overall, physicians may consider utilizing PDL for refractory AEGCG, although careful selection of PDL parameters should be made with individual considerations in mind such as risk of hyperpigmentation, lesion characteristics including size and depth, conditions predisposing to photosensitivity, and treatment goals. AEGCG is rare and difficult to treat, necessitating further research and increased reporting to broaden therapeutic options. Our report is limited by lack of follow-up beyond 5 months.

## Conflicts of interest

None disclosed.
